# GLTM: A Global-Local Attention LSTM Model to Locate Dimer Motif of Single-Pass Membrane Proteins

**DOI:** 10.3389/fgene.2022.854571

**Published:** 2022-03-15

**Authors:** Quanchao Ma, Kai Zou, Zhihai Zhang, Fan Yang

**Affiliations:** ^1^ School of Communications and Electronics, Jiangxi Science and Technology Normal University, Nanchang, China; ^2^ Artificial Intelligence and Bioinformation Cognition Laboratory, Jiangxi Science and Technology Normal University, Nanchang, China

**Keywords:** single-pass membrane protein, dimer motif, Bi-LSTM network, self-attention mechanism, motif localization model

## Abstract

Single-pass membrane proteins, which constitute up to 50% of all transmembrane proteins, are typically active in significant conformational changes, such as a dimer or other oligomers, which is essential for understanding the function of transmembrane proteins. Finding the key motifs of oligomers through experimental observation is a routine method used in the field to infer the potential conformations of other members of the transmembrane protein family. However, approaches based on experimental observation need to consume a lot of time and manpower costs; moreover, they are hard to reveal the potential motifs. A proposed approach is to build an accurate and efficient transmembrane protein oligomer prediction model to screen the key motifs. In this paper, an attention-based Global-Local structure LSTM model named GLTM is proposed to predict dimers and screen potential dimer motifs. Different from traditional motifs screening based on highly conserved sequence search frame, a self-attention mechanism has been employed in GLTM to locate the highest dimerization score of subsequence fragments and has been proven to locate most known dimer motifs well. The proposed GLTM can reach 97.5% accuracy on the benchmark dataset collected from Membranome2.0. The three characteristics of GLTM can be summarized as follows: First, the original sequence fragment was converted to a set of subsequences which having the similar length of known motifs, and this additional step can greatly enhance the capability of capturing motif pattern; Second, to solve the problem of sample imbalance, a novel data enhancement approach combining improved one-hot encoding with random subsequence windows has been proposed to improve the generalization capability of GLTM; Third, position penalization has been taken into account, which makes a self-attention mechanism focused on special TM fragments. The experimental results in this paper fully demonstrated that the proposed GLTM has a broad application perspective on the location of potential oligomer motifs, and is helpful for preliminary and rapid research on the conformational change of mutants.

## Introduction

Single-pass membrane proteins are one of the most widely classified membrane proteins, composed of a single transmembrane ^™^ helix and several water-soluble domains, and play an important role in cell signaling, motility, and material transport (Rawlings 2016). Compared with the active state of the multi-pass membrane protein is located within the TM helical bundle, the single TM helix of single-pass membrane protein was initially considered as a merely hydrophobic anchor (Zviling et al., 2007). However, the TM helix of single-pass membrane protein has been verified in making crucial contributions to the protein-protein interaction in recent years.

The intramembrane helix-helix interaction of single-pass membrane protein was firstly confirmed in the dimerization process of human glycophorin A (GpA). In the 3D model for the homo-dimer of human GpA, researchers observed the most helix contact points occurred in the GxxxG motif of TMD (Russ and Engelman 2000). Moreover, the statistical result indicated that the GxxxG motif was one of the significant expression residue pairs in the TM domain (Senes et al., 2000), and these single-pass membrane proteins have a high homo-dimerization tendency when their TM domain contains GxxxG motif (Brosig and Langosch 1998). Except for the GxxxG motif, the polar residue and the leucine zipper also confirmed their irreplaceability in the assembly of oligomeric complexes (Li et al., 2012). The interhelical hydrogen bond of the polar residue directly influences their dimerization degree (LaPointe et al., 2013). The leucine zipper is a ${\left(abcdefg\right)}_{n}$ heptad repeat motif with leucine at every fourth position and hydrophobic residues at every first position. This “knobs-into-holes” type of side-chain packing facilitates self-associates of the TM domain (Oates et al., 2010). Significantly, the conformational change of single-pass membrane protein as typically receptor activation basis selectively regulated cellular signaling (Hubert et al., 2010). Many diseases are directly related to the dysfunction of transmembrane receptor proteins, research of oligomers offers the opportunity to design drug targets and develop new pharmaceuticals (Cymer and Schneider 2010).

The amino acid residues frequency of the TM domain was used to distinguish different homo-oligomer forms in the earliest oligomer prediction model (Song and Tang 2005); their prediction results confirmed the importance of residue composition for protein quaternary structures. To avoid losing important sequence context information of protein sequence, the pseudo-amino acid composition (PseAAC) was proposed to replace the simple amino acid composition (Zhang et al., 2006). Discrete wavelet transformation was used to decompose digit signals of protein primary structure into different coefficients, and screen out effective global context features (Qiu et al., 2011). This global feature description method combined with a decision-tree algorithm obtained outstanding prediction accuracy (Sun et al., 2012). Moreover, the functional domain was discovered to be involved in molecular evolution in recent years. The functional domain information has been confirmed to improve the prediction performance, but the application of these oligomer prediction models was limited in the poor interpretability. For single-pass membrane proteins, an interpretability motif discovery approach was employed to locate their potential oligomer motifs by corresponding oligomer prediction results.

In previous functional motif detection studies, researchers mainly adopted rigorous statistical formulation to search for overexpression subsequence patterns (Liang et al., 2012). TMSTAT directly calculated the frequency of all pairs and triplets of residues to screen out overexpression subsequence patterns in the TM domain (Senes et al., 2000). A regular expressions algorithm was used to more precisely specify special residues position and interval size in SLiMFinder (Edwards et al., 2007). As researchers realized the complexity of nearby residues dependence, Markovian models were gradually used to discover potential motif patterns, such as NestedMICA (Dogruel et al., 2008), weighted hmm (Song and Gu 2015), and HH-MOTiF (Prytuliak et al., 2017). Note that these oligomer motifs as biologically defined anchors or landmarks are limited in a sequence interval. The discriminative motif discovery models DEME (Redhead and Bailey 2007) and DlocalMotif (Mehdi et al., 2013) introduced spatial confinement scores of each subsequence pattern to distinguish unrelated subsequence patterns and local functional motifs. DiMotiF proposed peptide-pair encoding (PPE) to probabilistic segmentation variable-length subsequence patterns and screened out positively related subsequences as potential motifs after annotating possible secondary structures of these subsequences (Asgari et al., 2019). Although these above search algorithms have strong statistical analysis ability to detect subtle subsequence pattern signals from large datasets, these motif discovery approaches cannot define their corresponding biological function for discovered subsequence patterns.

In this paper, we propose a motif localization model called GLTM to locate potential dimer motifs in the dimer prediction process. The Global-Local Bi-LSTM structure was the fundamental component of our motif localization model, and this idea of bilayer structure referred to the influence of highly conserved subsequence patterns and TM domain context information on oligomerization. Combined with the advantage of a Global-Local structure and the character of one-hot encoding, GLTM achieved a new data enhancement on the data preprocessing module. Additionally, new positional penalization was proposed to encourage a self-attention mechanism focused on known subsequence patterns. In the benchmark dataset, GLTM reached 97.5% accuracy and successfully located most key residue with self-focus and position penalization. Moreover, we discuss the existing deficiencies and application prospects of the motif localization model in the dimerization study of residue mutations.

## Materials and Methods

### Dataset

The Membranome database was the first comprehensive resource on single-pass membrane proteins and is widely used to assist analysis and computational modeling of single-pass membrane protein and their complexes (Lomize et al., 2017). The Membranome database collects and compiles diverse data of single-pass membrane proteins, including amino acid sequence, domain architecture, protein topology, and oligomeric states. More importantly, Membranome contains known key residues involved in the homo-dimerization interface according to both mutagenesis studies and computational models.

A new benchmark dataset was established and used for training and testing our motif localization model. Firstly, 334 homo-dimers, which were verified by nuclear magnetic resonance (NMR), mutagenesis experiments, crystal structures of dimers, or other validation methods of TM helix association, were collected from Membranome. Secondly, the orthologs of these 334 homo-dimers with similar oligomerization tendencies were collected from UniProt. Thirdly, chosen dimer motifs were spatially confined in the TM domain, and the C-terminal region of the TM domain participated in helix-helix interactions. Forty residues length of dimer fragment and no-dimer fragment were intercepted from each collected single-pass membrane protein sequence. Finally, the R_1937_ benchmark dataset collected 524 dimer fragments, 1,413 no-dimer fragments, and 24 known motif positions based on 70% maximal identity.

### Construction of GLTM Model

In bioinformatics areas, machine learning models widely used k-mers as the protein sequences representation method. Fixed-length subsequences were segmented from the original sequence and regarded as units of biological sequences to encoding in the k-mers treatment method. However, the directly one-hot encoding for subsequence units ignores these strong coupling effects between different positions in the oligomer research of TM protein (Liang et al., 2012). This means that the representation method of short sequence fragments needs to intensify the context information of the TM domain for the oligomer prediction task. Hence, an improved k-mers treatment method was proposed to intensify the independence of every residue based on Global-Local Bi-LSTM bilayer structure.

**FIGURE 1:**
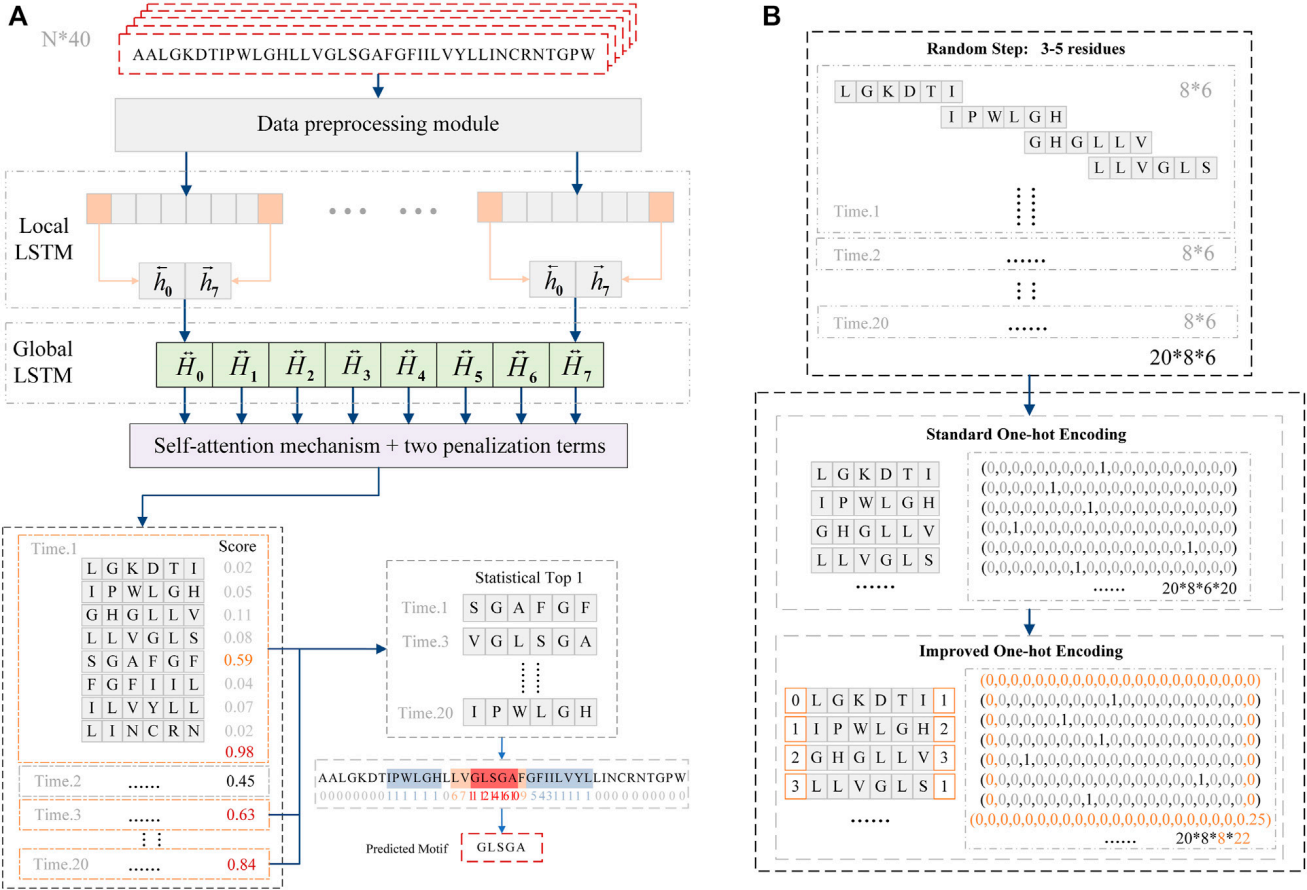
**(A)** GLTM model composed of data preprocessing module, local Bi-LSTM layer, global Bi-LSTM layer, and self-attention layer. After continuous twenty times prediction, the model chose the subsequence fragment that was predicted more than 10 times as potential motif. **(B)** Data preprocessing module used no-fixed step and improved one-hot encoding to encode original sequence fragment.

GLTM consists of the data preprocessing module, local Bi-LSTM layer, global Bi-LSTM layer, and self-attention layer (Figure 1A). The first data preprocessing module used the random step selection approach to segment the original sequence and used improved one-hot encoding to represent these repeated expression residues. Standard one-hot encoding used independent binary vector dimensions to respectively represent twenty standard amino acids (Jing et al., 2020). The K length of local subsequences was converted to a k*20 binary vector by standard one-hot encoding. Our improvement strategy takes advantage of the LSTM network, memory cell of LSTM accepts previous output and cell states as input, and transmits current output and cell states to the next memory cell, this Bi-LSTM structure effectively utilizes sequence context information. Referred to the idea of one-hot encoding, two new binary vector dimensions were proposed to represent repeatedly residues information between contiguous windows, and two window states were appended in every local window to represent repetitious residues numbers. Therefore, the bidirectional feature extraction process preferentially accepted repetition residues information on the local Bi-LSTM layer, and original local subsequences were encoded to k+2*22 binary vectors (Figure 1B).

After the data preprocessing module finished subsequences encoding, the encoded vectors directly input into their corresponding local window in the local Bi-LSTM layer. The next global Bi-LSTM layer only accepted the final state output of every local window to extract oligomerization features. Significantly, the weight redistribution process of the self-attention mechanism was the most critical function to locate motif. In order to redress these false weight redistribution processes, new penalization terms were proposed and applied in the last self-attention layer.

### Two Penalization Terms in Attentional Mechanism

The self-attention mechanism was widely applied in deep learning, and the redistributive weight of subsequence represented its importance degree for prediction results. Hence, in our motif localization model GLTM, the highest weight of local subsequence was regarded as the potential oligomer motif. When well-trained, GLTM had high prediction accuracy in recognizing dimer fragments. However, accurately locating motifs was always difficult in our previous experiments. This underlying problem, named shortcut learning, is a common deep learning symptom. Shortcut learning typically shows that the deep learning model usually chooses unintended features in prediction results without restricted conditions. Position penalization and self-focus penalization terms were proposed to reduce these fault localization of unintended subsequence patterns.

$$A\left(x\right)=softmax\left(W_{s2}\tanh\left(W_{s1}H{\left(x\right)}^{T}\right)\right)$$ (1)

GLTM randomly chooses n local window numbers from each sequence fragment, and the feature number of a local window is set as u in each unidirectional. Global Bi-LSTM hidden state H(x) is a weight matrix with a shape of n -by- 2u . The calculation of annotation vector A(x) needs to set an arbitrary hyperparameter $d_{a}$ . The weight matrix $W_{s1}$ is sized $d_{a}$ -by- 2u , and the matrix $W_{s2}$ has the shape 1 -by- $d_{a}$ . The softmax(∗) ensures all elements of annotation vector A(x) sum up to 1.

$$\begin{array}{cc} s_{i}=\frac{e^{c-\;\left|cen\left(x,i\right)-l\left(x\right)\right|}}{\sum_{n}e^{c-\;\left|cen\left(x,j\right)-l\left(x\right)\right|}}, & if\;l\left(x\right)\neq \emptyset \end{array}$$ (2)

$$S\left(x\right)=\;\left(s_{1},s_{2},\cdots s_{n}\right)$$ (3)

The window position score vector S(x) of these known dimer motifs was calculated in the data preprocessing module. Symbol c is an arbitrarily constant parameter, cen(x) represents the window center-positive of corresponding local subsequence, and l(x) is the center of these known oligomer motifs.

$$P\left(x\right)=\left\{ \begin{array}{l} {\left\|A\left(x\right)A{\left(x\right)}^{T}-I\right\|}_{2}^{2},if\;s=\emptyset \\ {\left\|S\left(x\right)-A\left(x\right)\right\|}_{2}^{2},if\;s\neq \emptyset \end{array}\right.$$ (4)

$$L\left(\theta \right)=\arg\underset{\theta }{\min}\left(\sum_{i=1}^{m}\left({\left\|y_{i}-sigmoid\left(A\left(x_{i},\theta \right)H\left(x_{i},\theta \right)\right)\right\|}_{2}^{2}+\alpha P\left(x_{i},\theta \right)\right)\right)$$ (5)

Self-focus penalization term enhances single-window weight by minimizing the disparity between $A\left(x\right)A{\left(x\right)}^{T}$ and an identity matrix. Position penalization is used to learn known motif distribution by minimizing the disparity between annotation vector A(x) and window position score vector S(x) for these known dimer motifs.

## RESULTS AND DISCUSSION

### Visualization Result of 26 Known Dimers

**FIGURE 2:**
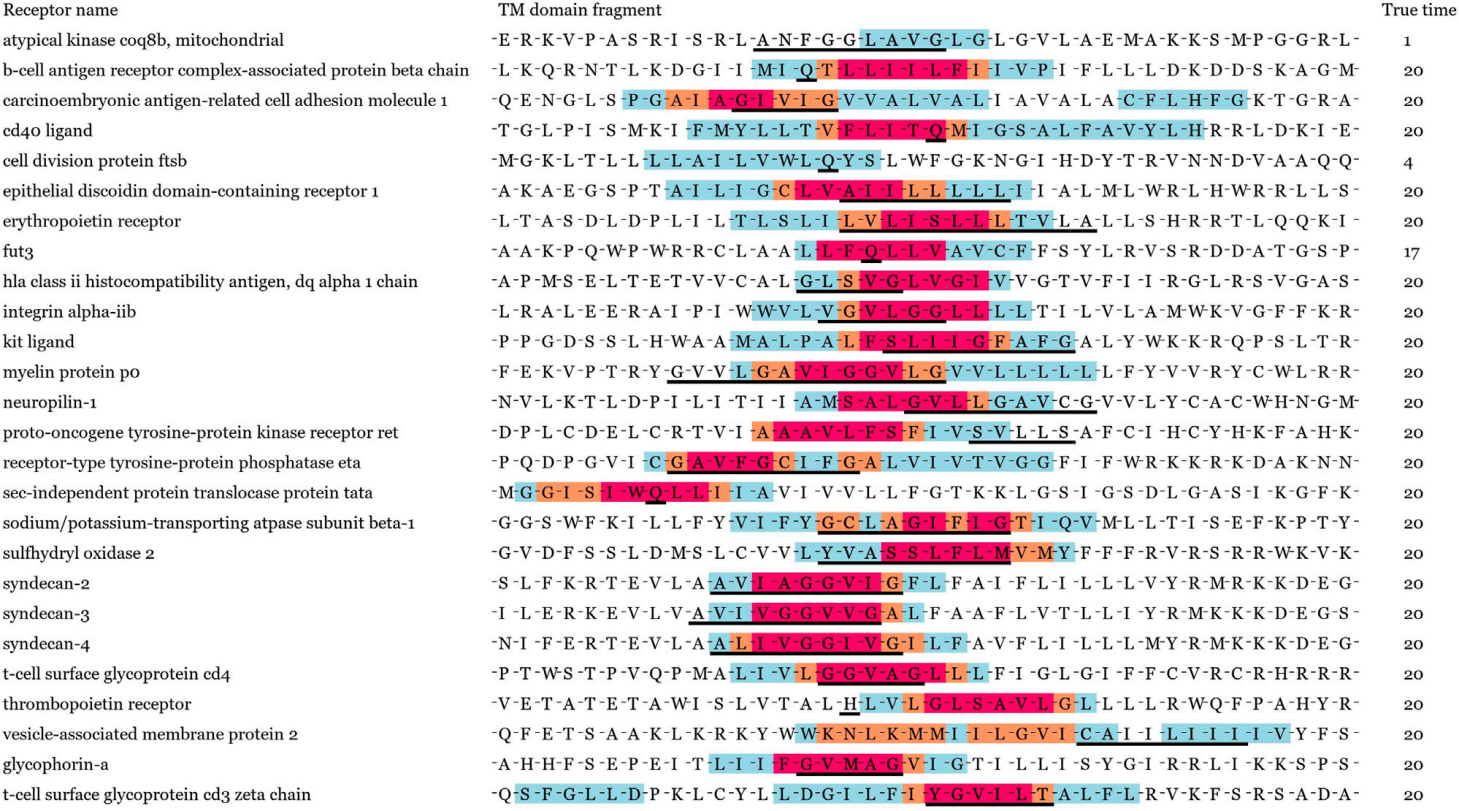
Visualization result of 26 known dimers. True time represents the total true prediction number in twenty prediction results. Black solid line shows key residues for known dimerization process. Different levels of potential motifs have been labeled in red, orange, and blue, respectively. The red denotes to the most important residues in dimerization.

In order to verify our model performance, we visualized prediction results and localization results for these containing key residues sequences in Figure 2. Note that the same sequence fragment has hundreds of digital matrix representations in the encoding stage. GLTM chose the highest weight local subsequence as a predicted dimer motif when this sequence representation was predicted to dimers and repeated this process twenty times to obtain the more robust localization result. Three color regions were used to mark different localization degrees for the dimer motif, the blue region represents that a subsequence has been predicted to be a dimer motif, the orange region represents more than five predictions as a dimer motif. The subsequences with the most robust prediction result, predicted more than 10 times, comprise the red region. These key residues involved in known dimerization are signalled by a black underline.

We show the prediction performance of GLTM with the different window size and number parameters in Table 1, and three evaluation indices were both more than 90% in all experiments. Most known key residues were steadily located in visualization results, in particular for the GxxxG motif of glycophorin A and YxxxxT motif of ζζ which belong to these overexpression subsequence patterns. Only mere unconventionality motifs were successfully located. It may cause by the scarcity of special dimer samples, and this guess was repeatedly verified in the following experiments.

**TABLE 1:** Accuracy performance of the model with different window size and window number.

Window number	5 residue lengths			6 residue lengths			7 residue lengths		
	Accuracy	Precious	Recall	Accuracy	Precious	Recall	Accuracy	Precious	Recall
9	0.966	0.934	0.942	0.971	0.936	0.956	0.969	0.932	0.954
10	0.96	0.921	0.932	0.974	0.956	0.947	0.971	0.938	0.954
11	0.961	0.926	0.928	0.965	0.934	0.936	0.974	0.942	0.96
12	0.975	0.94	0.968	0.973	0.929	0.969	0.974	0.945	0.959

### Effect of Two Penalization Terms

In previous experiments, we discovered these successfully located motifs lower than a quarter of the known key residues. In order to enhance the localization accuracy, we proposed two penalization terms to reduce mislocated subsequences, one was self-focus penalization, and the other was position penalization. The self-focus penalization was proposed to distinguish the critical local subsequence in the weight redistribution process. However, diversified oligomer motif localization only relied on self-focus penalization was insufficient. Position penalization was used to encourage the local window weight distribution to approximate the corresponding motif position distribution for these known dimer motifs.

**FIGURE 3:**
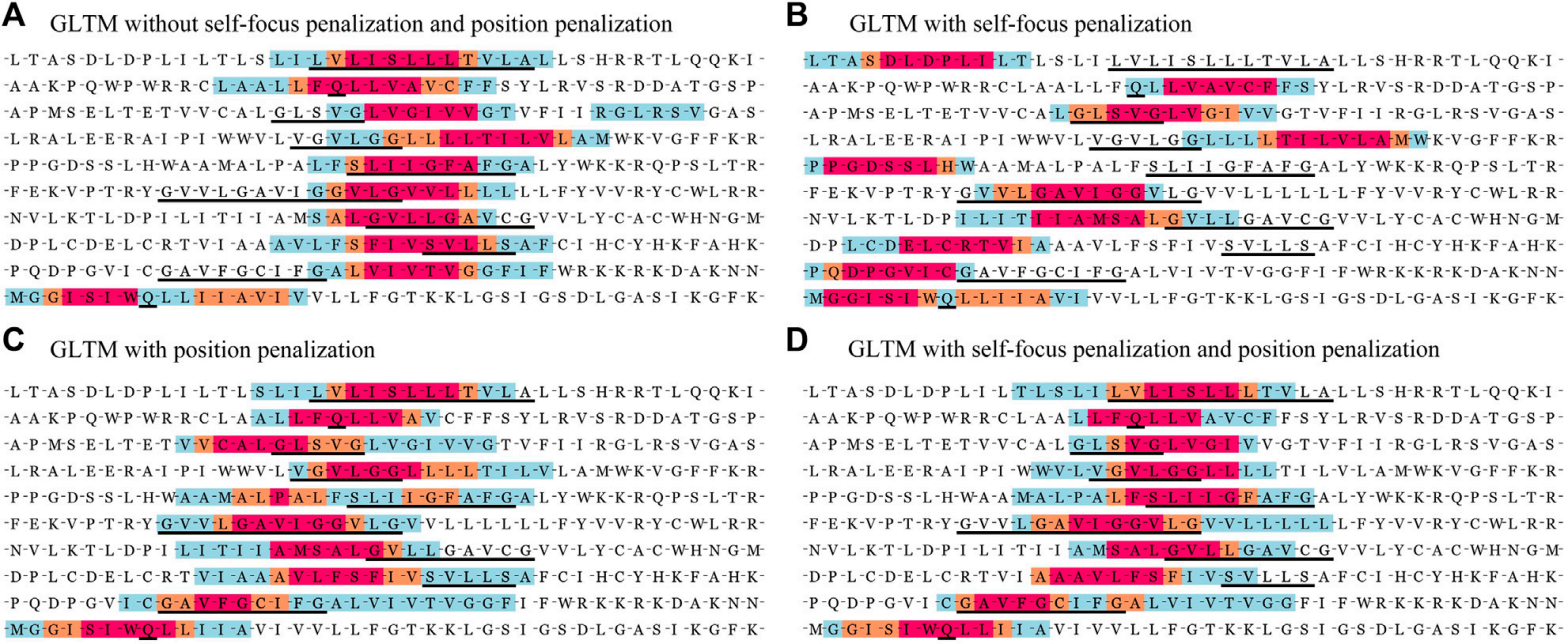
Part localization results of GLTM with different penalization combination.

**FIGURE 4:**
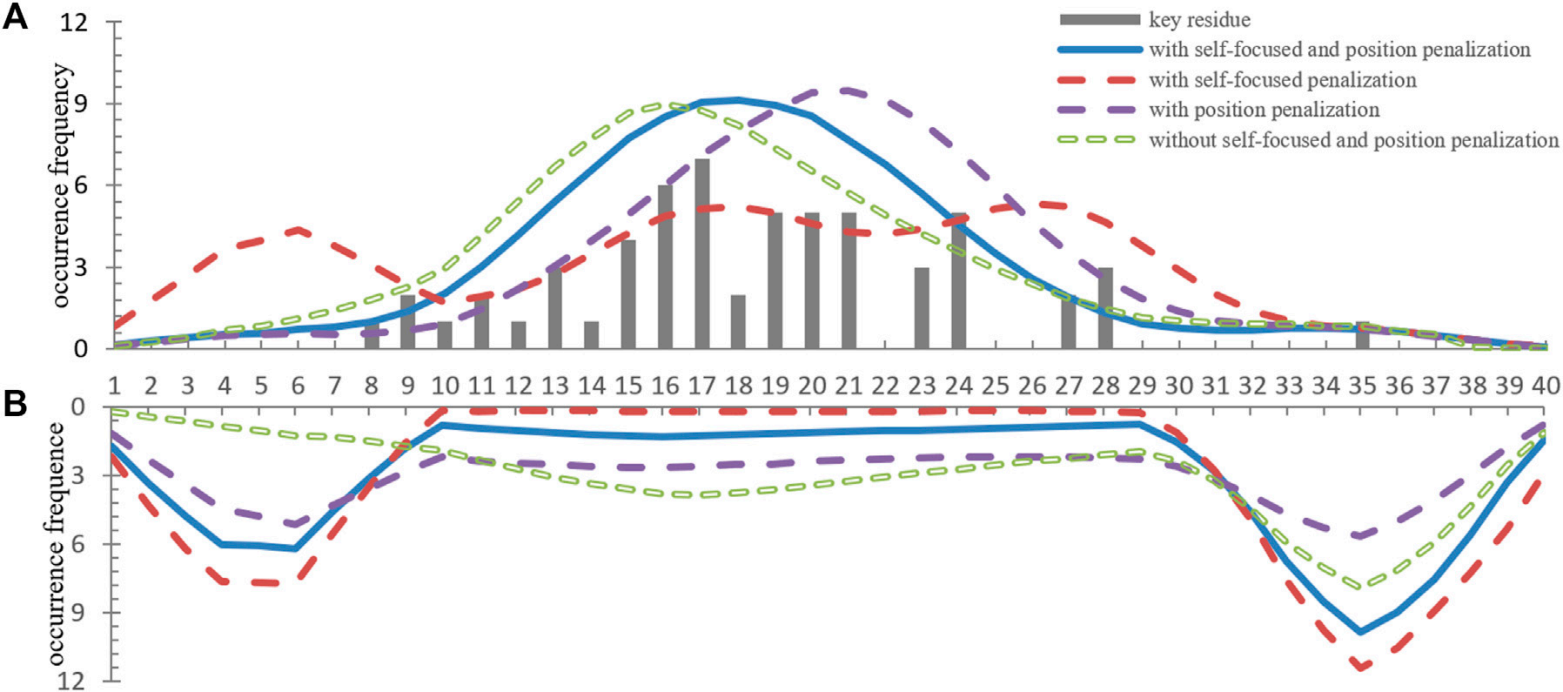
**(A)** The position distribution of located subsequences for dimer fragments. **(B)** The position distribution of high weight subsequences for no-dimer fragments.

In order to compare the localization performance with different penalization combinations, we showed the localization results of part known dimer sequences in Figure 3. Moreover, we drew the located subsequences position distribution of these dimer fragments and no-dimer fragments in Figure 4. Obviously, without self-focus penalization and position penalization, the located subsequence distribution for dimer fragment and no-dimer fragment had the same crest position. This means that the weight redistribution process focused on the specific position information rather than subsequence patterns. This tendency deviated from our oligomer motif localization principle. Two penalizations were both successfully reduced the unintended feature extraction for specific position information. However, part end-terminal subsequences were mislocated as potential motifs only with self-focus penalization. With self-focus and position penalization, GLTM reaches outstanding localization accuracy and stability in motif localization tasks.

### Dimer Motif Localization of TNF Receptor Superfamily

The tumor necrosis factor receptors superfamily (TNFRSF) is one of the most important single-pass membrane protein families. Most TNF receptors are candidates for antibody-based immunotherapy. A recently growing number of studies showed some tumor necrosis factor receptors play an active role in receptor signaling. In driving signaling, dimerization is an essential process which participates in the assembly of higher-order structures (Pan et al., 2019). In recent dimerization research, part potential dimer motifs of TNFRSF were speculated by alignment of TNFRSF sequences from various organisms (Zhao et al., 2020). These speculated dimer motifs referenced to prior biological knowledge had high credibility.

**FIGURE 5:**
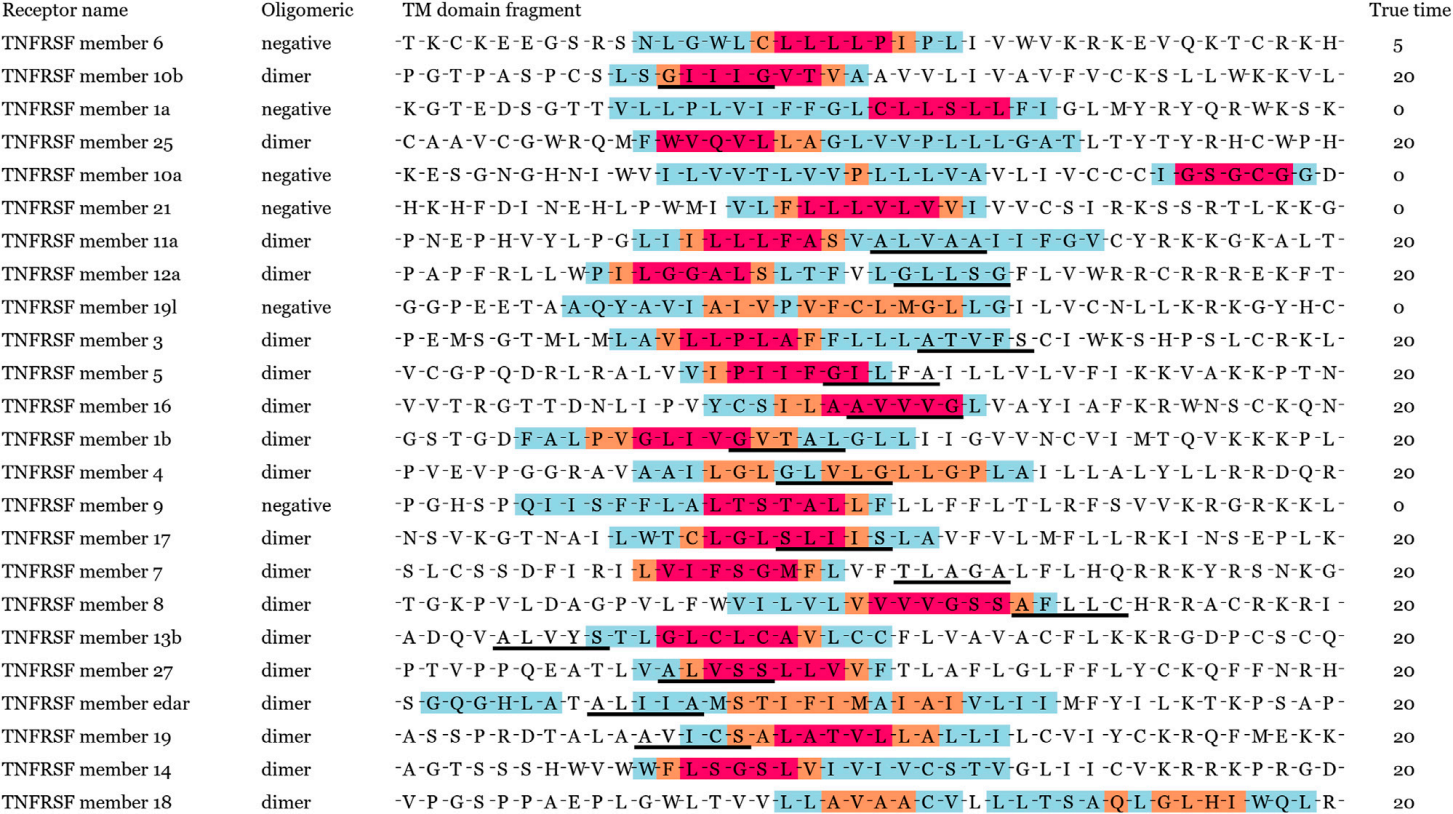
Different levels of potential motifs has been predicted and labeled in red, orange and blue, respectively. Red denotes to the core of the potential motifs. The speculative motifs generated by alignment of homologous species are marked by black solid line for comparison.

In order to verify our motif localization performance in the TNFRSF dataset, these TM sequences of TNFRSF were collected from UniProt version 2020_10. In the prediction results, partial TM sequences were falsely predicted to dimerize, and these subsequences of high weight were also marked in Figure 5. False prediction results were caused by the whole hydrophobicity discrepancy between training samples. Moreover, we noticed the most speculated dimer motifs was the GxxxG motif for TNFRSF, the known subsequence patterns information of the polar residue and the leucine zippers influenced specific GxxxG motif localization in position penalization.

**FIGURE 6:**
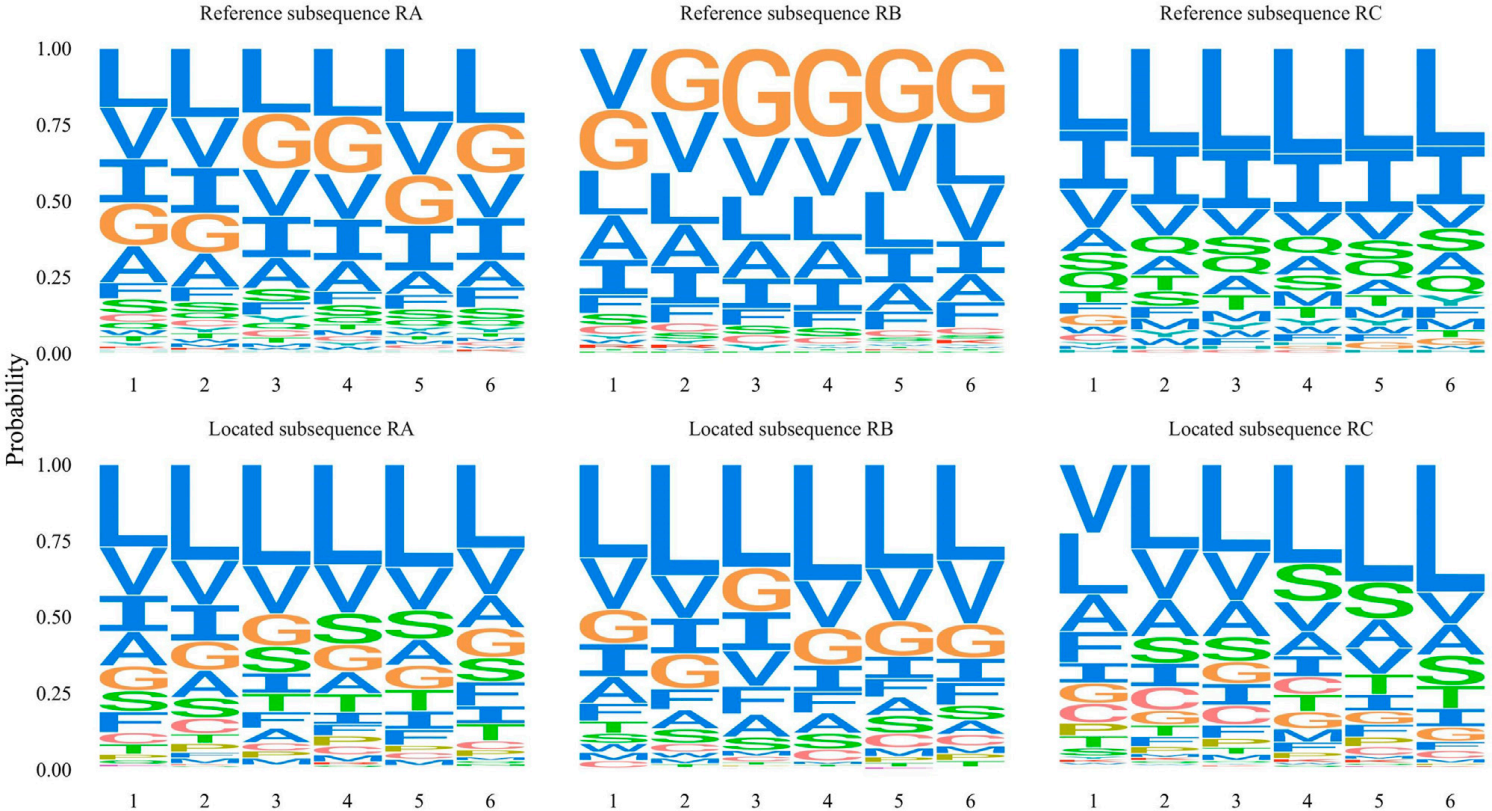
Three contrast experiments respectively used RA, RB, and RC training datasets which include different known motifs information. Corresponding reference subsequence represents the residues frequency of high window score subsequences. The located subsequence represents the frequency of 20 amino acid residues for all located subsequences.

We designed contrast experiments to verify the localization effect of position penalization. We set three new training datasets that include the different known motifs’ information. The RA dataset included the information of the known GxxxG motif, the polar residue, and the leucine zippers. The RB dataset only utilized the information of the known GxxxG motif, and the RC dataset had the information of polar residue and leucine zippers. High position score subsequences were collected from the training set, and their residue occurrence frequency was calculated as the reference subsequences in Figure 6. The located subsequences represented the residue occurrence frequency for these located subsequences. Besides these originally richly “blue” residues, the position penalization enhanced the specific motif localization performance according to supplied motif information.

### The Influence of Sequence Context for Its Dimerization

Oligomer motifs were usually simplified as a helix-helix interactions paradigm, but more and more studies have certified that these subsequence frames cannot simply be regarded as a surrogate tool for oligomer state determination (Li et al., 2012). Other residues also influence helix-helix interactions besides oligomer motifs. For instance, the TM domain context highly determines the thermodynamic stability of TM helix-helix interactions than local GxxxG motif in glycophorin A (Bano-Polo et al., 2012). The SDS-PAGE analysis of glycophorin A mutants demonstrated that the C-terminal region residues were also important for their helix packing (Bano-Polo et al., 2012). Partial residues deletion and replacement will damage oligomerization to different degrees (Orzaez et al., 2000). Moreover, researchers guessed the distance between the dimerization motif and the flanking charged residues play a key role in the stability of TM helix-helix interactions. We chose 17 sequence fragments to research oligomerization based on previous residue mutation experiments of glycophorin A and ζζ. The first fifteen sequence fragments had confirmed their dimerization degree in previous biological experiments, and the dimerization interface of the last seven mutants was destroyed by residue replacement.

**FIGURE 7:**
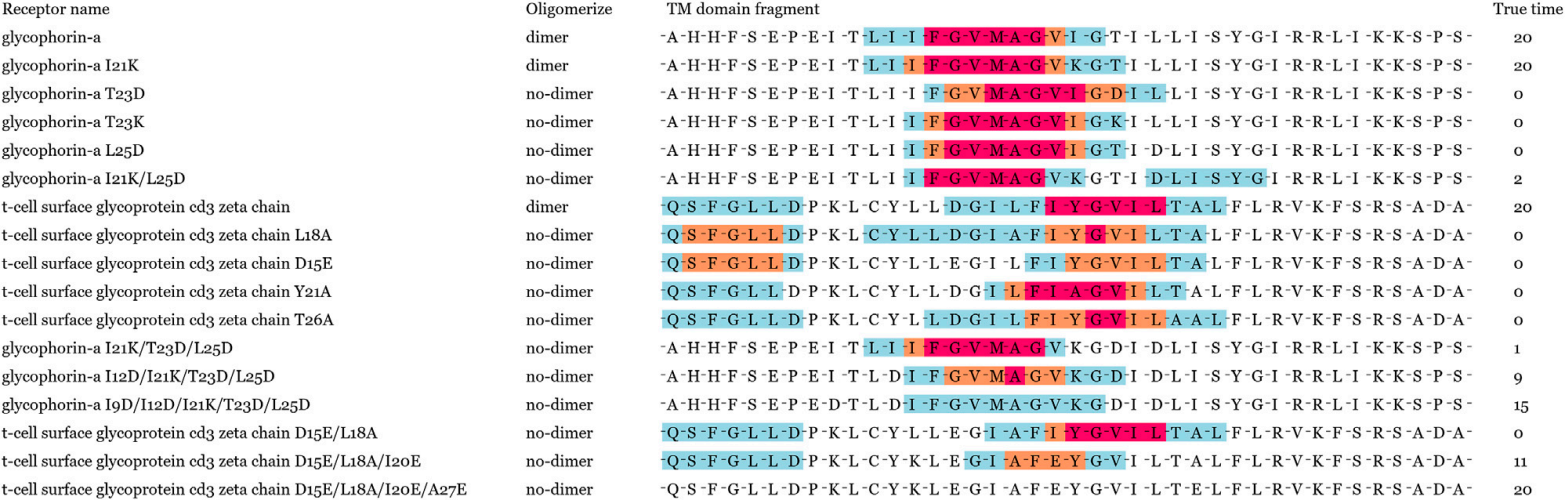
Visualization results of 17 mutants. The labels of first 11 mutants were confirmed in biological experiments, and the labels of last six mutants were speculated to be by their destroyed dimerization interface.

Most mutants of single hydrophobic residue replacement were predicted to dimerize in Figure 7. Although the prediction results of single residue mutants differ widely from the actual dimerization degree, other mutants were successfully predicted to not dimerize when the hydrophobic residues had been massively replaced. Significantly, the GxxxG motif and YxxxxT motif were stable when located in most mutants. This visualization results demonstrated that GLTM captured these overexpression subsequence patterns and considered sequence context information in oligomer prediction. Current experiments were limited in the lack of oligomer data. The motif localization model has broad application prospects in mutant oligomerization research with the rapid growth of sequencing data.

## CONCLUSION

In this paper, we propose an attention-based Global-Local structure Bi-LSTM model named GLTM to locate potential dimer motif. The three main components of GLTM can be summarized as follows: The first component was data preprocessing module, this module improved one-hot encoding to achieve a new data enhancement approach of subsequence segmentation; The secondary global-local Bi-LSTM structure was proposed to respectively extract local subsequence patterns and global context features; Proposed position and self-focus penalization reduce these irrelevant subsequences localization in tertiary attention mechanism layer. GLTM successfully located the most known key residues in the established benchmark dataset. In comparative experiments, the visualization results demonstrated the effectiveness of our proposed position and self-focus penalization. Different from the oligomer motif discovery method, our motif localization model achieved end-end motif localization function without multiple homologous sequences alignment. More importantly, our motif localization model has broad application prospects in the research of mutant oligomerization.

## Data Availability

The original contributions presented in the study are included in the article/Supplementary Material, further inquiries can be directed to the corresponding author.
